# Major Challenges in Rheumatology: Will We Ever Treat Smarter, Instead of Just Harder?

**DOI:** 10.3389/fmed.2019.00144

**Published:** 2019-06-26

**Authors:** Vasco C. Romão, João Eurico Fonseca

**Affiliations:** ^1^Department of Rheumatology, Centro Hospitalar Universitário Lisboa Norte, Hospital de Santa Maria, Lisbon Academic Medical Centre, Lisbon, Portugal; ^2^Rheumatology Research Unit, Instituto de Medicina Molecular, Faculdade de Medicina, Universidade de Lisboa, Lisbon, Portugal

**Keywords:** rheumatology, personalized medicine, stratification, rheumatoid arthritis, biomarkers

“*[A reply to letters recommending remedies]: Dear Sir (or Madam): I try every remedy sent to me. I am now on No. 67. Yours is 2,653. I am looking forward to its beneficial results*.”

Mark Twain, quoted in *My Father Mark Twain*, by Clara Clemens

## The Treatment Revolution in Rheumatology

The field of rheumatology has witnessed astonishing progress in the understanding and management of rheumatic diseases since the second half of the twentieth century. The discovery and introduction of glucocorticoids and conventional synthetic disease-modifying antirheumatic drugs (csDMARDs) into the therapeutic armamentarium of rheumatologists enabled, for the first time, to effectively change the natural course of disease and improve most clinical outcomes (1). The new millennium pushed the revolution further at an exponential level with the advent of sophisticated, biologically-engineered drugs—the so-called biologicals or bDMARDs—that targeted specific molecules in key pathogenic pathways and dramatically modified the prognosis of most patients with immune-mediated rheumatic diseases (2).

This progress, which was driven by tremendous research efforts to better understand the complex mechanisms behind each disease, has been particularly remarkable in inflammatory joint diseases such as rheumatoid arthritis (RA) and spondyloarthritis (including ankylosing spondylitis and psoriatic arthritis), and slower in the area of connective tissue diseases (e.g., systemic lupus erythematosus, Sjögren's syndrome) and vasculitis. Indeed, as of March 2019, 10 original bDMARDs with 5 different mechanisms of action are approved in Europe for the treatment of RA, 9 for psoriatic arthritis (4 mechanisms of action), 6 for ankylosing spondylitis (2 mechanisms of action), only 1 for systemic lupus erythematosus and small-vessel vasculitis and none for Sjögren's syndrome (3).

Yet, despite these significant advances, major unmet needs endure. The case of RA is paradigmatic of the current challenges faced by rheumatologists and patients alike in daily clinical practice. While at first, and especially when paralleled to other rheumatic diseases, RA seems to be the lucky relative of the rheumatology family with a variety of innovative bDMARDs available for treating and modifying the disease and improving patients' lives and outcomes, in practice the reality is more complex (4, 5).

## “*Me-too*” Drugs and the *Trial and Error* Approach

Firstly, after the major breakthroughs shown around the turn of the millennium by the pioneer bDMARDs approved for RA (infliximab and etanercept) in comparison to the standard of care available at the time (csDMARDs), the following decade observed a surge of other drugs that demonstrated a comparable effect in similar populations of patients (6). With a few exceptions (e.g., tocilizumab and sarilumab exhibiting superiority over methotrexate in monotherapy), new coming therapies usually conveyed a “*me-too*” effect that though important to increase treatment options in the event of inefficacy or intolerance, did not generate as tremendous an impact as its predecessors (7).

Secondly, the wide diversity of bDMARDs and modes of action contrasts with the profound lack of reliable, reproducible clinical and biological markers to inform treatment selection. Indeed, in spite of all the notable progress seen so far, we are somewhat surprisingly unable to recognize beforehand which individual patients will benefit more from a given drug, which will not respond at all and which are at a higher risk of toxicity or intolerance (8). Taking the specific example of RA, it should be acknowledged that there are a few well-established prognostic indicators that are associated at a group level with treatment-resistant disease, including female gender, older age, long lasting disease, failure of previous biologics, smoking, and high baseline disability (8, 9). But these features seem to be generically associated with worse treatment outcomes as a whole, rather than constituting specific predictors of response to a given drug. A couple of exceptions exist, such as the role of rheumatoid factor / anti-citrullinated protein antibodies seropositivity in determining a better response to rituximab (10) and abatacept (11) but also here this is a group effect and some seronegative patients will still show improvement with these treatments, while other seropositive patients will not experience any benefit. Other variables such as relevant comorbidities (e.g., lymphoma or monoclonal gammopathy) or infectious risk may further concede slight preference of one bDMARD over another and thus aid in the treatment decision process (12, 13), although again this is not driven by a particularly strong factor that identifies the best treatment for a given patient. This current landscape has inevitably led to the so-called *trial and error* approach that is the hallmark of present treatment strategies in RA and other inflammatory joint diseases and which has significant implications in terms of cost, risk and, ultimately, outcome.

## Limitations of Present Treatment Modalities

Undeniably, coupled with the major benefits brought by these therapies, a few shortcomings have emerged. These are powered by the aforementioned unprecise treatment paradigm, with implications both at the patient and societal level. The first factor is related to the significant direct costs associated with these drugs, which has put additional financial pressure in already struggling healthcare systems (14). However, it has been shown that the overall cost associated with RA management has not increased significantly over the last decades, due to a major drop in indirect costs and productivity losses that compensated for the higher drug-related expenditure (14, 15). In fact, the main concern is that lacking robust personalized treatment strategies, patients may be treated with costly bDMARDs for an extended period of time without experiencing any relevant benefit but still be exposed to its risks and potential adverse events. It is remarkable that in such a case, the risk-benefit ratio is clearly tilted in the wrong direction, and yet, health authorities, physicians and patients, all seem to ignore or accept this fact as inevitable.

Currently, bDMARDs have a well-established safety profile (16), that needs to be balanced against the corresponding benefits provided by the treatment itself. A number of serious conditions—such as tuberculosis and other serious infections or liver and medullary toxicity, to name just a few (17)—are associated with bDMARDs and are accepted only in return for substantial efficacy and improvement of short- and long-term outcomes. If this second part of the equation is missing, as is the case of the considerable proportion of patients that fail to see any benefit at all, it may be ethically (and financially, as explained above) unacceptable to prescribe and administer these drugs. Hence, the problem relies in the fact that we are unable to identify these patients beforehand, emphasizing the limitations of this treatment model and the need for an individualized approach. The scenario is aggravated when we also take into account the short-term, highly-intensive, remission-inducing regimens that are applied in several rheumatic diseases, usually with substantial toxicity, in an indiscriminate manner (18–20). These treatment modalities represent the standard of care, but personalized treatment could revolutionize the current paradigm of an all-or-nothing approach simply based on the existence of a certain diagnosis.

Another aspect that should be considered when analyzing the issue of undiscerning drug selection is effective treatment delay. Treat-to-target (T2T) approaches have shown that, in terms of prognosis, more important than the drug administered is the therapeutic target defined and the quickness to attain it (21, 22). Subjecting patients to treatments that will not be effective for long periods—at least 3 to 6 months as per standard recommendations (23)—will cost precious time during which disease activity is high and structural damage readily occurs. This leads to poor long-term outcomes and is yet another reason for why a generalized same-drug-for-all strategy is flawed. The discovery of precise biomarkers of response to inform treatment selection could save up this lost time and, thus, synergistically reinforce the T2T strategy. In spite of this, T2T advocates have, somewhat surprisingly, disregarded the importance of personalized medicine vs. the main goal of abating disease activity regardless of the mechanism implied and drug chosen (24). However, as they point out, this only reflects the current standing, where precise biomarkers that have a major impact on treatment selection and can modify and guide clinical practice are still missing (8, 25).

Importantly, one should not forget other additional factors contributing to treatment limitations. Despite major improvements in the area of early diagnosis, it has recently been reported that in daily clinical practice the reality is still far from optimal (26–28). Moreover, there has also been a continuous global effort for the development and update of classification criteria of rheumatic diseases, but these are aimed at patient recruitment in research studies, in most cases perform poorly in a real-world setting, and therefore should not be applied for clinical diagnosis (29). Finally, with the incorporation into routine care of highly sensitive diagnostic techniques such as ultrasonography or magnetic resonance imaging, the concern of overdiagnosis and overtreatment of rheumatic diseases is already a reality, that should be addressed (30). These aspects allow to better understand the delicate landscape in which drugs are prescribed and underscore the need to improve treatment approaches.

## New Players: The Role of Biosimilars and Novel Targeted Synthetic Molecules

As we have exposed, currently available bDMARDs compose an heterogeneous group of drugs, with several modes of action, distinct dosages, schedules, and routes of administration and some particularities in terms of concomitant medication, monitoring, or adverse events. However, the overall efficacy and safety between bDMARDs is considered to be roughly similar and long-term outcomes of patients treated with these drugs are not substantially different (2, 7). For this matter, we should highlight the importance of disease registers, both national and international, which have greatly contributed to demonstrate the benefits and pitfalls of treatments in a real-life setting (31).

It is in this setting that in the last 5–10 years two new treatment classes have appeared to add to the complexity of rheumatic patients management: biosimilar DMARDs (bsDMARDs or biosimilars) and targeted synthetic DMARDs (tsDMARDs). Both have contributed to widen the options available for treating RA patients, but also brought along additional challenges to the table.

Biosimilars emerged following the patent expiry of bDMARDs and promised to increase patient access by significantly decreasing treatment costs while, simultaneously, showing comparable efficacy and safety (32, 33). Following rigorous clinical trial programs demonstrating equivalence to the original bDMARDs, there are currently 16 bsDMARDs approved in Europe for the treatment of RA (4 infliximab, 3 etanercept, 6 adalimumab, 3 rituximab), with others awaiting approval (2 adalimumab), already withdrawn (2 adalimumab) or not having applied for RA indication (3 rituximab) (3). These impressive numbers speak well to the potential impact of bsDMARDs in the field. Indeed, its main added value relies in the reduced cost-−20 to 40% below reference bDMARDs, depending on country—and, consequently, the larger number of patients that can be treated with these drugs (33, 34). While this partially resolves one of the issues mentioned above (cost), the other two (safety and time lost) remain unchanged. Ultimately, the increase in offer could even amplify the problem, with patients switching often between different bDMARDs and bsDMARDs in the pursuit of the right drug, with the associated implications in treatment delay and pharmacovigilance issues. This further reinforces the need for patient stratification and rational treatment selection.

Nonetheless, bsDMARDs have undoubtedly opened a new era in the treatment of rheumatic diseases. Rates of first bsDMARD are rising in Europe (34), and after the main pivotal trials, good quality observational data have confirmed the safety of switching patients from the original drug to its biosimilar (35–37). Concurrently, other challenges arise, such as selection and switching between biosimilars of the same bDMARD, different immunogenicity patterns and, potentially, lack of evidence for established prognosis markers that may differ from those known for the original drug (35, 36). This latter aspect may be fueled by a low willingness of bsDMARD drug developers to better explore disease heterogeneity, as this could potentially be commercially unattractive and limit the promotion of these drugs. Additionally, the *nocebo effect*—the negative effect of a treatment that is attributable to poor patient expectations—is a well-defined phenomenon that is particularly troublesome when switching real-world patients from original bDMARDs to bsDMARDs, due to the importance played by subjective measures (e.g., pain and global assessment, tender joint counts) in the evaluation of disease activity and treatment response (38).

A novel class of oral highly specific small molecules inhibiting intracellular signaling pathways, the tsDMARDs, has also become available (39). Tofacitinib, a Janus kinase inhibitor approved in the United States (2012) and Europe (2017), was recently followed by baricitinib (2018 and 2017, respectively) as the first two oral drugs that have an efficacy and safety profile comparable to bDMARDs (40). This is a major advance, given the preference of many patients for oral vs. parenteral administration. Other potential advantages include rapid clinical efficacy, even in monotherapy, absence of immunogenicity and a shorter half-life, facilitating the management of adverse drug reactions (39, 40). However, its place in treatment algorithms (before or after cs/b/bsDMARDs) is still to be fully understood. Most importantly, while tsDMARDs will definitely be beneficial for a large number of patients, the lack of predictive biomarkers precludes its rational application at the individual patient level and its introduction in the clinical armamentarium follows the same trial and error approach.

## The lag of Personalized Medicine in Rheumatology

A number of reasons can be put forward as to why personalized medicine is taking a long time to materialize in rheumatology. First, the heterogeneous and multifactorial nature of immune-mediated rheumatic diseases, with complex pathogeneses, makes it unlikely that a single marker of a given pathway will discriminate response of several different DMARDs with contrasting modes of action (41). Second, a considerable amount of effort is dedicated to identifying biomarkers in the blood, far from the key immunopathologic events happening at the synovial tissue, which may prove more informative (42). Third, one aspect that is not so commonly cited relates to the subjective nature of a significant part of the tools used to assess treatment response, remission status or disability. This applies both to the patient (e.g., visual analog scale) and the physician (e.g., joint counts) and is, by definition, influenced by many other individual-related factors, such as personality, previous experience with a given drug, expectations, patient-doctor relationship, cultural context, comorbidities, etc. (43, 44). Indeed, this scarcity of hard outcomes contrasts to that seen, for instance, in the area of oncology (e.g., death, tumor-free survival), where personalized treatment has long been a reality. To what extent is the current situation explained by this fact is unclear, but subjective measures are likely to play an important role in confounding study results, potentially leading to the loss of a weak, albeit unique signal.

## Conclusions and Future Perspectives

In summary, the present moment in rheumatology is an exciting one, after two fast-paced decades that transformed the prognosis of patients with inflammatory rheumatic diseases. This was mainly due to a deep expansion of available, effective therapies that have come, nonetheless, coupled with major challenges that need to be tackled. We argue that this is the time to do so, where research efforts should be best directed at establishing robust biomarker-based treatment models that will allow individualized care. If successful, the outcome of this approach is likely to translate into more substantial benefits, compared to the meek pursuit of new drugs—often with the same or close mechanisms of action—that will provide a similar overall effect to currently available options. Synovial tissue should be at the center of these investigations, as targeting the disease process at its core will arguably prove most valuable. This is definitely a sinuous path, not without many expectable setbacks, but one worth tracking as its completion may finally lead to a new longed-for era of personalized medicine in rheumatology. Notably, despite all the cutting-edge science behind these innovations, clinical expertise of rheumatologists will be of strategic importance in guiding the process along the way.

## Author Contributions

VR and JF contributed to manuscript conception and design, literature review, manuscript preparation, and critical review. Both authors have read and approved the final version of the manuscript.

### Conflict of Interest Statement

The authors declare that the research was conducted in the absence of any commercial or financial relationships that could be construed as a potential conflict of interest.
